# A New Bioactive Fibrin Formulation Provided Superior Cartilage Regeneration in a Caprine Model

**DOI:** 10.3390/ijms242316945

**Published:** 2023-11-29

**Authors:** Elif Vardar, Hui Yin Nam, Ganesh Vythilingam, Han Ling Tan, Haryanti Azura Mohamad Wali, Eva-Maria Engelhardt, Tunku Kamarul, Pierre-Yves Zambelli, Eleftheria Samara

**Affiliations:** 1 Pediatric Orthopedic Department, Children’s Hospital, Chémin de Montétan 16, 1004 Lausanne, Switzerland; elfvardar@gmail.com (E.V.); eva.engelhardt@epfl.ch (E.-M.E.); pierre-yves.zambelli@chuv.ch (P.-Y.Z.); 2 Tissue Engineering Group, Department of Orthopaedic Surgery (NOCERAL), Faculty of Medicine, Universiti Malaya, Kuala Lumpur 50603, Malaysia; huiyin26@yahoo.com (H.Y.N.); hantan6188@gmail.com (H.L.T.);; 3 Nanotechnology and Catalysis Research Centre (NANOCAT), Universiti Malaya, Kuala Lumpur 50603, Malaysia; 4 Pediatric Surgery Unit, Department of Surgery, Faculty of Medicine, Universiti Malaya, Kuala Lumpur 50603, Malaysia; ganesh@um.edu.my; 5 Animal Experimental Unit, Faculty of Medicine, Universiti Malaya, Kuala Lumpur 50603, Malaysia; azurawali@um.edu.my

**Keywords:** articular cartilage, functional tissue regeneration, insulin-like growth factor 1, fibrin

## Abstract

The effective and long-term treatment of cartilage defects is an unmet need among patients worldwide. In the past, several synthetic and natural biomaterials have been designed to support functional articular cartilage formation. However, they have mostly failed to enhance the terminal stage of chondrogenic differentiation, leading to scar tissue formation after the operation. Growth factors substantially regulate cartilage regeneration by acting on receptors to trigger intracellular signaling and cell recruitment for tissue regeneration. In this study, we investigated the effect of recombinant insulin-like growth factor 1 (rIGF-1), loaded in fibrin microbeads (FibIGF1), on cartilage regeneration. rIGF-1-loaded fibrin microbeads were injected into full-thickness cartilage defects in the knees of goats. The stability, integration, and quality of tissue repair were evaluated at 1 and 6 months by gross morphology, histology, and collagen type II staining. The in vivo results showed that compared to plain fibrin samples, particularly at 6 months, FibIGF1 improved the functional cartilage formation, confirmed through gross morphology, histology, and collagen type II immunostaining. FibIGF1 could be a promising candidate for cartilage repair in the clinic.

## 1. Introduction

Articular cartilage lesions are one of the most common and challenging problems in the knee joint and affect millions of people worldwide. Differences in the severity of cartilage lesions were found in 63% of knee diagnostic arthroscopic surgical procedures, and they can affect approximately 12% of the population at any age [1]. A genetic predisposition and acute and repetitive trauma, including accidents, sports injuries, and arthroscopic procedures, can cause serious focal-area damage to the articular cartilage. Mechanical symptoms (e.g., catching and instability), effusion, and a restricted articular range of motion are the most common symptoms associated with damaged cartilage. The choice of the most appropriate treatment modality for cartilage defects is subject to the severity of the symptoms and the damage degree. In most cases, microfracture and mosaicplasty are the most applied surgical procedures in the clinic [2]. Microfracture is an operative technique based on creating multiple micro-channels in the subchondral bone, inducing the infiltration of mesenchymal stem cells (MSCs) to the damaged site to trigger regeneration. In mosaicplasty or osteochondral autograft transfer (OATS), the defect area is filled with small bone–cartilage plugs harvested from non-weight-bearing regions of the knee. Although the short-term clinical results of these two techniques seem to be satisfactory, they mostly result in the recurrence of symptoms and fail to trigger functional tissue formation in the long term [3,4]. 

Avascular cartilage tissue has no direct connection to the lymphatic and vascular systems, which are unable to trigger an inflammatory response immediately, thus limiting its capacity for self-regeneration. To address this significant challenge, the use of cell-based approaches has been favored in cartilage defect treatment since the late 1980s [5]. Autologous chondrocyte implantation (ACI) is a reliable technique for patients having defect sizes of more than 2.5 cm^2^ and having a sufficient amount of cartilage tissue around the defect site [6]. ACI has a three-step treatment process, consisting of the harvesting of patients’ chondrocytes from the knee joint, the ex vivo expansion of chondrocytes, and the implantation of these cells back into the defect site. To secure the implant, a periosteal flap is sutured onto the defect site to prevent the injected cells from leaking. However, challenges such as donor morbidity, an unwanted host immune response, the risk of contamination, and the potential problems associated with storage and transportation represent limiting factors in the widespread clinical acceptance of utilization [7]. Another promising approach is the local injection of mesenchymal stem/stromal cells (MSCs) into the damaged cartilage. Human mesenchymal stem cells have the innate ability to differentiate into different tissue types, including cartilage, bone, and muscle. In contrast to ACI, MSCs based approaches have several benefits, including the higher ex vivo expansion capacity and lowering the risk of cartilage biopsy-specific morbidity [8]. In several preclinical studies, the intra-articular injection of exogenously expanded MSCs effectively hindered cartilage degradation and joint inflammation [9,10]. In the study of Kim et al., the efficacy of human synovium-derived MSCs was validated in a canine osteoarthritis (OA) model [11]. The results showed that human synovium-derived MSC injection into the injured joints significantly enhanced cartilage regeneration and decreased inflammation at the defect site. On the other hand, one of the main limitations related to the clinical transition of MSC injections is method standardization to induce and maintain a stable chondrocyte-like cell population, leading to functional hyaline cartilage regeneration. 

During the past few years, biologically active biomaterials have shown great potential in tissue regeneration for tissue engineering applications. The restorative effect of several growth factors, such as bone morphogenetic protein 2 (BMP-2), platelet-derived growth factor (PDGF), vascular endothelial growth factor (VEGF), fibroblast growth factor 2 (FGF-2), and insulin-like growth factor 1 (IGF-1), has been shown in many recent papers [12,13,14,15,16]. In articular cartilage, several cytokines and growth factors, such as IGF-1, act in signaling pathways to regulate the homeostasis of articular cartilage. In particular, IGF-1 is one of the proteins that modulates MSC chondrogenesis, triggering the up-regulation of chondrocyte markers [17]. IGF-1 also affects the phenotypic and morphologic responses of chondrocytes [18]. In this study, we propose a bioactive fibrin injectable, conjugated with recombinant IGF-1 (FibIGF1), as a functional treatment option for cartilage lesions. Compared to our previous study [19], the FibIGF1 formulation was reformulated, which might facilitate its speedy transition to the market. Moreover, this new formulation was tested for cartilage regeneration, a new indication, which would broaden the intended use of FibIGF1 in the clinic. 

Platelet-rich plasma (PRP) is autologous and concentrated plasma containing various types of cytokines and growth factors derived from patients’ blood [20]. These proteins play a critical role in chondrocyte apoptosis, differentiation, remodeling, and inflammation [21]. Moreover, several other molecules released by platelets also trigger the production of collagen II and fibrin, acting as a chemo-attractant scaffold for stem cells to migrate into injured sites, leading to functional tissue regeneration [22]. In a meta-analysis study, thirty-four randomized controlled trials were evaluated in terms of the efficacy of PRP treatment in comparison to other intraarticular treatments in patients with knee OA [23]. The results showed that the patient-reported outcomes based on the Western Ontario and McMaster Universities Osteoarthritis Index (WOMAC) subscores (pain, stiffness, and function) were significantly better after 12 months. On the other hand, the burst release of the bioactive factors is one of the key challenges in PRP therapy, resulting in repeated and painful injections for patients requiring long-term treatment [24]. The defective cartilage needs mechanical support during the healing process. According to an interesting study, the regeneration potential of a novel plasma-poor growth-factor-rich hyaline cartilage chip was assessed in full-thickness cartilage defects in the medial femoral condyles of sheep [25]. The preliminary results showed improved chondrogenesis, functionally regenerated hyaline cartilage, and normal macroscopic ICRS assessment 6 months after the surgery. Thus, the use of growth factors could provide an alternative treatment to articular cartilage repair in the clinic due to their capability to promote regeneration and repair. 

At present, there is not yet a fully effective clinical solution for patients with cartilage defects. As such, the development of different techniques is the focus of a great deal of research. Thus, in this study, the stability, safety, and functional regeneration capacity of a new bioactive fibrin formulation was assessed in a full-thickness cartilage defect in a caprine model. The bioactive fibrin consists of recombinant IGF-1 covalently conjugated to fibrin microbeads. The clinically relevant caprine model was used to investigate the potential of this recombinant IGF-1 to trigger related cell infiltration and differentiation at the defect site. The findings of this study could lead to improvements in the understanding of cartilage repair and subsequently lead to the better application of tissue engineering in the repair of cartilage defects in clinical practice.

## 2. Results

### 2.1. Stability and Sterility of Fibrin Formulation

**Figure 1:**
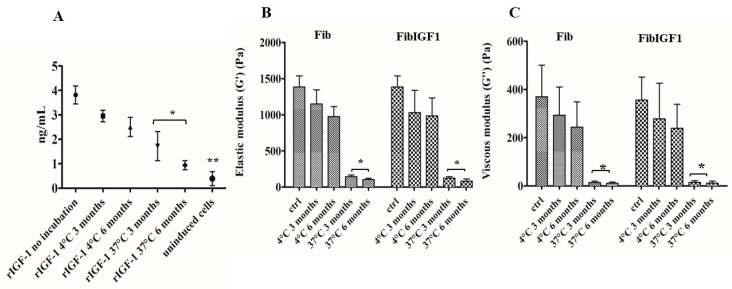
Stability test results. (**A**) IGF-1 receptor phosphorylation assay demonstrated that both recombinant IGF-1 and the samples that were stored at 4 °C significantly induced the IGF-1 receptor phosphorylation of bovine chondrocytes as compared to uninduced cells (*n* = 3, ** *p* < 0.002). The biological activity of recombinant IGF-1 was hindered when the samples were stored for 3 months at 37 °C (*n* = 3, * *p* < 0.03). (**B**) Elastic modulus (G′) and (**C**) viscous modulus (G″) values of fibrin microbead (Fib) and recombinant IGF-1-loaded fibrin (FibIGF1) were determined at a constant frequency of 1 Hz. The prepared fibrin samples were used as control samples (ctrl). Storing samples at 37 °C significantly decreased both the elastic and viscous modulus values of fibrin samples in comparison to control samples (*n* = 4, * *p* < 0.01).

A phosphorylation assay was used to determine the activity of rIGF-1 (ng/mL) in the fibrin samples, incubated at two different storage temperatures, 4 °C and 37 °C. For each time point, the activity of rIGF-1 was determined by quantifying the phosphorylated rIGF-1 receptors of chondrocytes using a sandwich ELISA assay. Twelve months after incubation either at 4 °C or 37 °C, fibrin microbead samples were degraded by plasmin. The degradation solution was used for the phosphorylation assays to evaluate the biological activity of rIGF-1 at different time points. At six months, the amount of phosphorylated rIGF-1 receptors from the samples stored at 4 °C was around 2.8 ng/mL, but this amount significantly decreased to 1.4 ng/mL for the samples stored at 37 °C (Figure 1A). Overall, there was no significant difference in the initial and final biological activity of rIGF-1 stored at 4 °C when compared to the control fibrin microbead samples that were freshly fabricated before the assay. 

**Figure 2:**
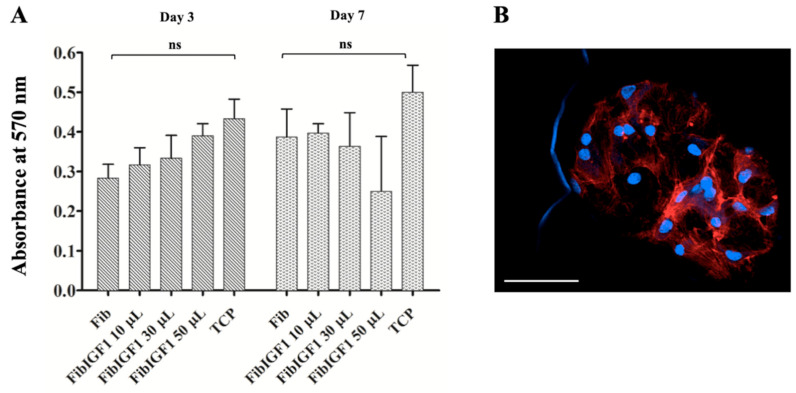
MTT assay results for bovine chondrocytes cultured with either fibrin microbead (Fib) or recombinant IGF-1-loaded fibrin (FibIGF1) for 7 days and fluorescent confocal microscopic image of bovine chondrocytes cultured with FibIGF1 on day 4. The metabolic activity of bovine chondrocytes on a tissue culture plate (TCP) was used as a positive control group. (**A**) Bovine chondrocytes were cultured using different amounts of FibIGF1. Data are expressed as mean ± SD based on three independent experiments with triplicate wells for each group. ns: non-significant. (**B**) Representative image of bovine chondrocytes on FibIGF1 samples. The bovine chondrocytes were stained with phalloidin for F-actin (red) and DAPI for cell nucleus (blue). Scale bar represents 50 μm, magnification 20×.

The oscillation rheological properties of the fibrin samples were studied to assess the mechanical stability of the fibrin samples for up to 6 months. At both 3 and 6 months, there was a slight decrease in both the viscous and elastic modulus values of fibrin samples stored at 4 °C (Figure 1B,C). Furthermore, as compared to freshly prepared fibrin samples, no change in either shape or size was observed with these samples during the incubation period of 12 months. In contrast, when the fibrin samples were stored at 37 °C, a significant difference between the initial and final elastic and viscous modulus values was observed from 3 to 6 months, as shown in Figure 1C and Figure 2B. The elastic modulus values of fibrin microbeads decreased 10-fold compared to control samples starting from 3 months at 37 °C.

The sterility of the fibrin samples was evaluated using FTM and SCDM culture media, validating the presence of anaerobic bacteria, aerobic bacteria, and fungi. After 28 days, optical density measurements showed that the culture media density was between 0.01 and 0.03 for all tested samples, whereas the minimum value for *E. coli* was obtained as 2.54. No color change was observed in the culture media of any sample set. Additionally, the fibrin samples were tested for bacterial endotoxins using LAL endotoxin assay kits. The obtained maximum concentration values from the LAL assay were below the threshold limit of 5 EU/kg, which was calculated using the dose limit calculations for drug products according to the European Pharmacopoeia policy on bacterial endotoxins [26]. 

### 2.2. In Vitro Culture of Fibrin Formulation

The MTT assay was used to evaluate the cytotoxicity of fibrin samples towards bovine chondrocytes. No significant differences in cell metabolic activity were observed between the tested groups containing 10 μL and 30 μL fibrin microbeads. However, increasing the FibIGF1 amount to 50 μL resulted in increased cytotoxic effects on the bovine chondrocytes on day 7. The human chondrocytes expanded over the surfaces of the fibrin microbeads starting at day 4 (Figure 2A). They created self-organizing chondrocyte networks on the microbead surfaces, which was confirmed using phalloidin and DAPI staining (Figure 2B). These results indicate that fibrin, either loaded with rIGF-1 or alone, favored bovine chondrocyte proliferation, adhesion, and expansion over 7 days. 

### 2.3. In Vivo Caprine Study

All goats recovered very well and were able to walk 2 h after the surgery. There was no sign of joint pain or impaired joint movement in any animal. Among all the operated goats, one of them presented mild swelling due to an infection in the knee in the first month following the procedure. Both 1 and 6 months after the surgery, the goats could fully place weight on the operated joints. No limping, swelling, or infection was observed by the end of the study. No deaths or serious adverse events were recorded.

**Figure 3:**
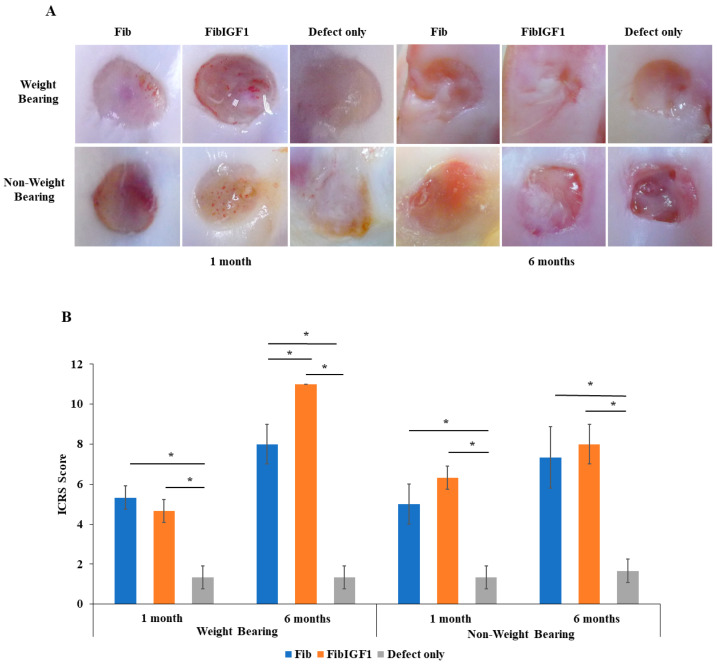
Macroscopic observation and the International Cartilage Repair Society (ICRS) scoring of chondral defect repair in each group at 1 and 6 months post-surgery. (**A**) Representative macroscopic images of weight-bearing and non-weight-bearing groups, with different cartilage healing treatments. (**B**) Macroscopic ICRS scoring of chondral defect repair. The group treated with either Fib or FibIGF1 had significantly increased scores compared to the defect group, with higher scoring in the FibIGF1 group (*n* = 3, * *p* < 0.05).

Goats were sacrificed at 1 and 6 months post-surgery. Cartilage regeneration was evaluated by the gross and microscopic analysis of the knee tissue 1 month and 6 months after treatment. Compared to the 1-month group, which showed partial filling of the defects, the macroscopic appearance of the FibIGF1-treated specimens demonstrated a marked improvement in the filling of the defects. The defects appeared to be almost filled, where the reparative tissue was seen to be similar to hyaline cartilage (Figure 3A). Light pink–white coloration was observed in the repaired tissue area compared to the surrounding host tissue. Significant differences in the ICRS scores (Figure 3B) were observed between FibIGF1 and the fibrin and chondral defects (*p* < 0.05). Cartilage healing with FibIGF1 treatment was more evident in the weight-bearing group compared to the non-weight-bearing group, particularly at 6 months.

**Figure 4:**
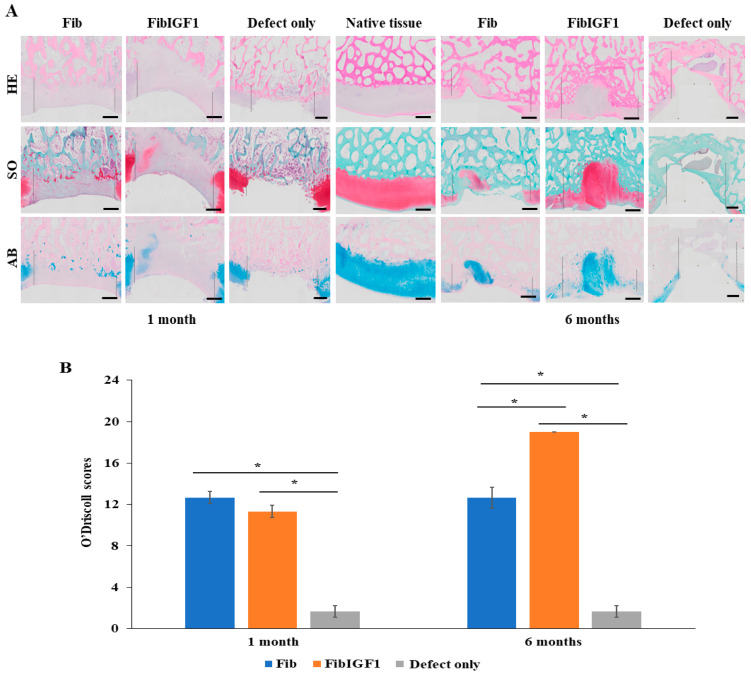
Histological results and O’Driscoll scores of cartilage defects in different groups at 1 and 6 months post-surgery. (**A**) Representative images of HE-stained sections of normal cartilage tissue at 1 month after surgery. No new tissue formation was observed with the defect-only sample. The arrows show the defect site. Representative images of HE-stained (purple chondrocytes), SO-stained (red proteoglycan, chondrocytes, and type II collagen), and AB-stained (blue glycosaminoglycans) sections of articular cartilage and subchondral bone tissue at months 1 and 6. Incomplete healing with fibrous tissue filling was observed in the defect site at 1 month post-operation. Compared to fibrin-only injection groups, more cartilage-like tissue was observed in the regenerated area at 6 months post-injection of IGF-1-loaded fibrin (FibIGF1). The images chosen from the defects created on the weight-bearing zones. The black dotted lines denote defect borders. Scale bars represent 1 mm, magnification 10×. (**B**) A bar graph illustrating macroscopic International Cartilage Repair Society scoring results (ICRS) of chondral defect repair. Significance is represented by * compared to other groups at same time point, compared to the same group at different time points (*n* = 3. Error bar = ± SD).

**Figure 5:**
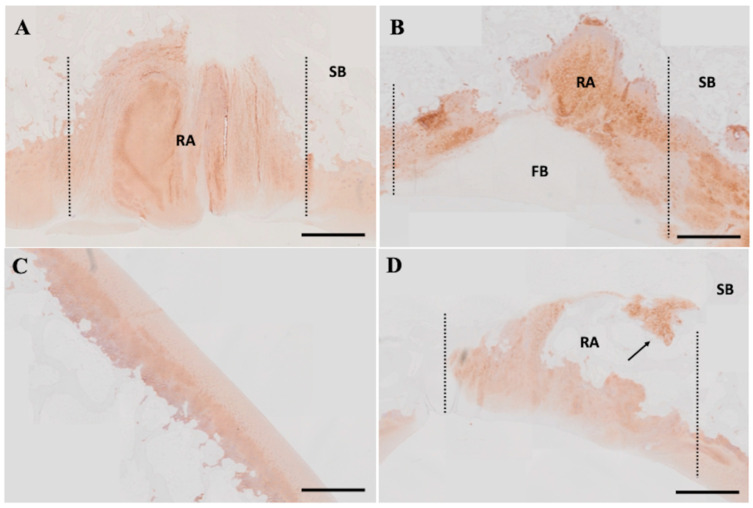
Immunohistochemical staining for type II collagen protein expression (brown) in the defect area for different groups. Abbreviations: Subchondral bone (SB), regenerated area (RA), and fibrotic tissue (FB). Representative images of type II collagen protein-stained section of (**A**) IGF-1-loaded fibrin (FibIGF1) injection sample, (**B**) fibrin-only injection sample, (**C**) normal cartilage tissue. (**D**) Host cell infiltration from the subchondral bone layer towards the defect area (shown with black arrow) at 6 months after surgery, specifically observed in FibIGF1 samples. The images chosen from the defects created on the weight-bearing zone. The black dotted lines denote defect borders. Scale bars represent 1 mm, magnification 10×.

Tissue samples from the weight-bearing group were chosen for further microscopic analysis based on the macroscopic results. In addition, regarding the microscopic results of H&E and Safranin O staining, cartilage regeneration in rIGF-1-treated defects was more evident in comparison to the Fib-only and defect-only groups at 6 months (Figure 4A). However, there was no difference between rIGF-1-treated and fibrin-treated defects at 1 month, indicating no full cartilage healing at earlier time points. The non-treated defects did not appear to undergo any healing over the 6-month period. In contrast, at 6 months, the rIGF-1-treated repaired tissue was hyaline-like cartilage tissue, with good integration with the surrounding tissue and a good thickness and surface regularity. The presence of proteoglycans and GAGs was confirmed by Alcian Blue staining (Figure 4A). The concentrations of these glycans also increased from the surface to the deep zone of the regenerated area. As healing was apparent in the 6-month group, collagen type II was further examined. The immunostaining results showed that cartilage-specific collagen type II expression was notable within the defect area for all tested materials 6 months after the surgery (Figure 5). Overall, the fibrin formulations, either plain or containing recombinant IGF-1, were integrated into the surrounding normal cartilage well. The modified O’Driscoll histological scores (Figure 4B) of the post-implantation repair tissue were significantly (*p* < 0.05) higher in the transplanted sites of the rIGF-1-treated group than for the group with plain fibrin and the untreated group. 

## 3. Discussion

The current study is an expansion of our previous studies in which we developed several bioactive fibrin formulations and assessed their regeneration capacity in different animal models [19,27]. In the current study, the new bioactive fibrin formulation (FibIGF1) was further improved, characterized, and tested in a clinically relevant caprine defect model to evaluate its regeneration capacity as an alternative treatment for full-thickness cartilage lesions. The study showed that FibIGF1 triggered accelerated cartilage regeneration at both 1 and 6 months compared to control samples (Group 3). 

The chemical, physical, and biological characterization of novel injectables is crucial for their smooth transition to the clinic. The main objective of the stability experiments was to determine for how long the fibrin formulations would remain biologically active and mechanically stable in the course of future clinical research. Science-based accelerated stability studies are used for the justification of the shelf life and storage conditions of products [28]. The stability of FibIGF1 was evaluated through rheology and ELISA experiments. Both human articular cartilage and fibrin show dynamic elastic and viscous properties; hence, they are viscoelastic materials [29]. A viscoelastic material can withstand applied stress for a period of time; it does not immediately flow. Upon removal of the stress, the material slowly recovers to its original state; however, the recovery is usually incomplete. Moreover, several recent studies have demonstrated that viscoelastic hydrogels could facilitate the regulation of stem cell phenotypes and chondrogenic differentiation [30,31,32,33]. Additionally, it has been shown that a significant elasticity increase is observed in native tissue after trauma or disease. Moreover, the native tissue continuously loses its ability to dissipate energy during fibrotic tissue formation [34]. Consequently, the related changes in native tissue elasticity and viscosity could modify cell matrix signaling pathways, resulting in the inhibition of stem cell migration and tissue repair-related gene and protein expression [35]. Unfortunately, the current cartilage repair techniques mostly result in fibrotic tissue formation, contributing to disease progression, although they initially provide spontaneous healing. 

At 3 months, the elastic and viscous moduli of FibIGF1 samples stored at 37 °C significantly decreased by more than 50% compared to the freshly prepared FibIGF1 samples. The addition of IGF-1 could hinder the interaction between the fibrinogen chains and thrombin during polymerization, resulting in dangling ends within the polymer chain segments. This might lead to crosslink imperfections during polymerization, resulting in a decrease in the mechanical properties of FibIGF1 at 37 °C [36]. Based on the results presented here, FibIGF1 can be safely stored at 4 °C for 6 months before use. 

The rIGF-1 activity was evaluated using the receptor phosphorylation assay at different time points. The samples incubated at 37 °C showed a significant decrease in the stimulation of chondrocyte receptor activity compared to freshly prepared FibIGF1 samples (Figure 2A). The significant declines in both the elastic modulus values and biological activity of the beads were attributed to potential fibrin degradation at 37 °C, which was triggered by unknown additives within the commercially available fibrin glue [37]. Thus, the fibrin’s degradation could have resulted in uncontrolled rIGF-1 removal from the FibIGF1 samples. The activity results are in agreement with the rheological findings. 

FibIGF1 was produced using a microfluidic platform, described in our previous studies [38]. In this study, the microfluidic production of fibrin microbeads was further modified to create standardized methods for product quality. After the microchips’ sterilization, they were coated with Sigmacote as an additional step, which might also introduce potential contamination to the system. It was observed that the bead production yield was significantly decreased without Sigmacote application. It has been shown that Sigmacote can be used for hydrophobic surface coating to facilitate stable bead formation [39]. Thus, a future study will address the removal of the Sigmacote step from our current methods. Two culture media, which were fluid thioglycollate medium (FTM) and soybean–casein digest medium (SCDM), were used to test the sterility of FibIGF1. FTM is used to detect aerobic and anaerobic microorganisms, and SCDM is used to detect aerobic bacteria and fungi [40]. The test samples were inoculated into the culture media and incubated at both 4 °C and 37 °C for 14 days. An *E. coli* (*Escherichia coli*) strain was used as a positive control. The results were evaluated by visual inspection and optical density measurements obtained via UV spectrophotometry (NanoDrop). The results of the sterility test on day 28 showed that all tested samples were free from contaminating microorganisms and were safe for further use. Additionally, larger amounts of bacterial lipopolysaccharide (LPS) contamination derived from recombinant growth factors are known as one of the major manufacturing risks, resulting in elevated local and systemic inflammatory responses as well as a potential delay in regeneration [41,42]. LAL assays are capable of detecting a range of endotoxins within the raw and final products [43]. The assay results showed that all tested fibrin formulations were below the threshold pyrogenic dose of endotoxins. Overall, both the sterility and endotoxin study results showed that FibIGF1 is potentially safe for animal and human use. 

Fibrin is a biocompatible and non-toxic natural polymer; however, manufacturing residuals such as surfactants and oil could be critical as they might evoke cytotoxic reactions. The MTT assay was conducted on bovine chondrocytes to assess the cytotoxicity of different fibrin sample groups. The MTT assay is a simple, versatile assay that complies with the requirements of biocompatibility testing for new materials [44]. Based on the cytotoxicity and DAPI/phalloidin staining results, both FibIGF1 and plain fibrin microbeads supported the growth, adhesion, and migration of the bovine chondrocytes. No significant metabolic activity changes were seen in bovine chondrocytes cultured with either FibIGF1 or plain fibrin compared with the TCP on days 1 and 7. On the other hand, the cell metabolic activity was slightly reduced in the FibIGF1 group compared to the TCP and plain fibrin groups on day 7. This decrease might be associated with chondrocyte phenotype maintenance at high cell seeding conditions [45]. Moreover, a large percentage of viable cells attached, migrated, and created a network on the fibrin microbeads, as confirmed by phalloidin/DAPI staining (Figure 2B). The chondrocytes maintained their round shapes with an evident actin network when they were cultured with the fibrin microbead samples. The results of this study indicate that all tested groups had good biocompatibility for cartilage repair. These results were expected because fibrin-based biomaterials have ideal biocompatibility and biodegradability and strong adhesion to biological surfaces [46].

The safety and functional regeneration capacity of FibIGF1 was assessed in a clinically relevant caprine model, which facilitates the validation of the potential products for further studies involving human subjects in the future. Both human and goat cartilage share similar characteristics, such as poor self-healing capabilities and comparable articular cartilage and subchondral bone organization [47]. Histopathological evaluations revealed no immune response that was triggered by the different fibrin formulations tested in the goat knees. Based on the 1-month in vivo results, the presence of a repaired matrix was evident at the injection site for both the plain fibrin and FibIGF1 groups (Figure 4). Moreover, all groups showed good integration with the host tissue at the boundary. One month after operation, a group of chondrocytes, having a large spherical morphology and stained positive by Safranin O, was observed at the native tissue junction within the repaired matrix tissue in the FibIGF1-treated group (Figure 4). The presence of these cells might be attributed to the chondrocytes’ hypertrophic process, resulting in endochondral ossification [48]. Additionally, the higher regeneration score for defect regions at the weight-bearing compared to non-weight bearing sites (Figure 3) could be explained by MSCs’ chondrogenesis, as confirmed by microscopic examination (Figure 4B). It is known that chondrocytes tend to increase the production of ECM components when they are exposed to mechanical loading [49]. Moreover, the chondrogenic differentiation of MSCs is triggered by the cellular environment when subjected to shear and compression forces [50]. This can be exploited with the concept of mechanotransduction, which shows that mechanical forces play a fundamental role in cell behavior and their adaptation to their environment [51], where it is particularly essential to maintain tissue homeostasis.

The articular cartilage protects joints from high friction and sudden shock loads. It has a dense extracellular matrix (ECM) composition, composed of mainly type II collagen. Type II collagen presents a specialized fiber network and provides resistance to strong and repetitive loads of compression, tension, and shear. Moreover, a high concentration of aggrecan proteoglycans (PGs) and glycosaminoglycan (GAG) is present in the ECM. Both PG and GAGs, which are the most abundant organic constituents of cartilage after collagen, regulate the cellular interaction with the neighboring cells, which determines the early cell fate during regeneration [52]. Thus, the presence of newly formed, proteoglycan-rich ECM tissue at the defect site is a strong indicator of functional regeneration [53]. For both the plain fibrin and FibIGF1 groups, 6-month histological analysis demonstrated an ECM composed of newly formed collagen fibers and proteoglycans at the defect area (Figure 4A and Figure 5). The positive staining of collagen type II in the regenerated area proved the presence of mature chondrocytes. As revealed in Figure 4 and Figure 5, both PG and collagen II fibers were continuously present at the regenerated area. The increase in PG and expressed collagen type II in the repair tissue revealed that rIGF-1 stimulated chondrogenesis. 

The FibIGF1 group exhibited better reconstruction of cartilage compared to both the fibrin and defect-only groups after 6 months. Moreover, it was observed that the regenerated articular cartilage zones in the FibIGF1 group showed a particular cellular arrangement similar to the native zonal organization of articular cartilage (Figure 4). The plain fibrin group showed mainly fibrous tissue filling, as depicted in Figure 3 and Figure 4. Several studies have suggested that exogenous IGF-I increases matrix proteoglycan and collagen type II deposition in vivo [14,54,55]. Collagen II expression areas were mainly concentrated in the deep layers of the regenerated area (Figure 5). This finding seems to agree with the study of Lorenzo et al., where the authors claimed that the cells in the deeper layer of articular cartilage favored the production of thicker collagen fibers [56]. Therefore, the higher collagen II expression in the FibIGF1-injected group might be associated with the differentiation of MSCs infiltrated from the bone marrow and local rIGF-1 release, which might stimulate chondrogenic differentiation. Moreover, many patients with arthritic cartilage show an increase in IGF-I levels at the early phase of OA, which might promote local tissue healing [57]. It has been also shown that IGF-1 downregulates the expression and secretion of degradative enzymes, such as collagenases, MMP-1, MMP-3, MMP-8, and MMP-13 [58,59]. Taking these findings together, local IGF-1 release might also contribute to the formation of neo hyaline cartilage-like tissue at the defect area. These results agree with previous studies demonstrating the dose-dependent regeneration potential of rIGF-1 [19,38]. Additionally, the tissue-adhesive ability of fibrin was not affected by the microfluidic production, as confirmed by our surgeons during the goat surgeries (Supplementary Video S1).

In this study, we developed a bioactive injectable as an alternative treatment option for patients with cartilage defects and cartilage lesions. A minimally invasive and non-repetitive treatment option could be a good alternative to current cartilage repair approaches. The stability results showed that FibIGF1 was stored safely for 6 months at 4 °C. In vivo results confirmed that FibIGF1 triggered a better cartilage regeneration outcome compared to the plain fibrin group. 

In conclusion, the histology findings demonstrated that FibIGF1 triggered significant cartilage regeneration at the defect site 6 months after the surgery. This promising approach could be an effective alternative treatment for patients with chondral defects. Thus, the use of biologics, such as recombinant growth factors, holds great promise as a future treatment option for cartilage lesions due to their ability to accelerate the rate and quality of tissue regeneration. However, the transition of biologics is challenging due to their structural complexity. Patients’ access to biological products could be made possible with a well-established development plan, tackling manufacturing and regulatory obstacles. A preclinical toxicity study for rIGF-1 is necessary to extrapolate the safe dose results to humans. A longer-term regeneration study is also needed to assess the long-term efficacy of FibIGF1.

## 4. Materials and Methods 

### 4.1. Animal Handling and Study Design

The approval of this experimental study was provided by the Institutional Animal Care and Committee (IACUC) in the Faculty of Medicine, Universiti Malaya (Ref. no.: M/03102019/25062019-02/R). All the experiments were conducted with strict adherence to the institutional guidelines for the care and use of laboratory animals in research. A total of 18 skeletally mature male Boer breed goats (*Capra aegragushircus*) were used, with an average age of 1.6 years (1.5–2.0 years) and average body weight of 32.0 kg (28.8 kg–35.0 kg). Each goat underwent a complete physical examination to ensure that it was healthy prior to inclusion in the study. The goats were group housed for one week to acclimatize to the housing conditions and were fed twice per day with mineral lick and hand-fed grain, followed by fasting for 12 h prior to surgery. The goats were divided equally into 3 groups that received different therapies: Group 1—fibrin microbead (Fib) treated; Group 2—recombinant IGF-1-loaded fibrin (FibIGF1) treated; Group 3—no treatment, i.e., defect only. The right knee was used as a reference, i.e., a normal knee. There were 2 time points to this study, 1 and 6 months, thus dividing the members of each group equally (*n* = 3/group).

### 4.2. Preparation and Characterization of Bioactive Fibrin Formulation

rIGF-1-loaded fibrin micro-beads were prepared using a microfluidic system, as previously described by Vardar et al. [38]. The microfluidic system was placed into a laminar flow hood during manufacturing to ensure sterility. The sterile microchips were obtained from EBERS (Spain). The fibrin beads were produced using a commercially available fibrin glue kit (Tisseel kit, Baxter). Briefly, the fibrinogen and the enzyme mix were transferred from the Tisseel kit to the vials. The solutions were then loaded into gas-tight glass syringes. The rIGF-1, which covalently binds fibrin, was produced by ExcellGene SA and then purified at École polytechnique fédérale de Lausanne (EPFL). Then, 30 μL of rIGF-1 was added to a syringe loaded with the enzyme before microfluidic bead production. The microfluidic system was operated at predefined flow rates, described in our previous papers [19,38]. After each production cycle, the beads were incubated at 37 °C for 40 min and then washed several times with phosphate-buffered saline (PBS). The excess water was removed using a cell strainer. A total of 100 μL fibrin was loaded into 300 μL insulin syringes using a spatula. The sterile syringes were stored at 4 °C. 

### 4.3. Stability and Sterility Experiments

The short-term and long-term stability of the fibrin sample beads was evaluated via structural, growth factor bioactivity, and mechanical property analyses. The change in the bioactivity of the rIGF-1 over time at both 4 °C and 37 °C was determined through a receptor phosphorylation assay (Human Phospho-IGF-I R/IGF1R DuoSet IC ELISA, DYC1770). The fibrin samples were degraded using plasmin before the activity experiments. The rIGF-1 concentration was determined using a BCA protein assay kit (Pierce™ BCA Protein Assay Kit, 23225) with lysates from degraded fibrin samples at different time points. The lysate solution was added to 6-well plates containing starving bovine chondrocytes (P5), which were extracted from mature bovine knee joints and expanded as described previously [60] to induce receptor phosphorylation. After 3 h, the cell lysate was placed in vials and stored at −80 °C for further use in the receptor phosphorylation assay, following the manufacturer’s instructions. The fresh fibrin samples produced before the activity experiments were used as a positive control group. To evaluate the mechanical stability, 200 μL of fibrin was stored in vials at either 4 °C or 37 °C for a year. The storage and loss moduli of the samples were measured using a C-VOR Shear Rheometer (Bohlin Rheometer, Germany) at months 6 and 12. A parallel plate geometry of 8 mm diameter was used. A constant oscillatory mode was applied to the samples with a frequency of 1 Hz, a temperature of 37 °C, and a gap distance of 2 mm throughout all tests. The average shear storage (G′) and loss (G″) moduli as a function of time were calculated over eight cycles for each hydrogel. 

The sterility of the fibrin bead samples was assessed using culture media, namely, fluid thioglycollate medium (FTM) and soybean–casein digest medium (SCDM). The test was a presence–absence test, in which the turbidity of the culture media was indicative of microbial growth. FTM was used to detect aerobic and anaerobic microorganisms, and SCDM was used to detect aerobic bacteria and fungi. The fibrin test samples, either with or without rIGF-1, were inoculated into the FTM and SCDM media and incubated at both 4 °C and 37 °C for 14 days. An *E. coli* strain (a kind gift from the Laboratory of Protein Design & Immunoengineering at EPFL) was used as a positive control. The results were evaluated by visual inspection and optical density measurements obtained from a NanoDrop spectrophotometer. Additionally, the limulus amebocyte lysate (LAL) assay (Pierce™ Chromogenic Endotoxin Quant Kit, A39552) was further performed to test endotoxin contamination in the purified rIGF-1. Briefly, different concentrations of rIGF-1 and control samples were mixed with the LAL reagent in a 96-well plate, and a Tecan plate reader was used to measure the absorbance at 405 nm. 

### 4.4. Cytocompatibility and Cell Adhesion Experiments

The cytocompatibility of the fibrin formulation was evaluated via 3-(4,5-dimethylthiazol-2-yl)-2,5-diphenyl tetrazolium bromide (MTT) assay (MTT Assay Kit, Abcam, ab211091) using bovine chondrocytes. First, 12-well plates were seeded with bovine chondrocytes (P5), which were extracted from mature bovine knee joints and expanded as described previously [60], at a seeding density of 1 × 10^5^ before fibrin addition. Four hours after cell seeding, various amounts of fibrin, including 10 μL, 30 μL, and 50 μL, were added into each well. The plates were cultured using DMEM supplemented with 10% fetal bovine serum (FBS, Gibco) and 1% PS for 7 days at 37 °C and 5% CO_2_. At days 3 and 7, 50 μL/well of MTT reagent was added to each well, and then the plate was incubated at 37 °C. After 3 h, the MTT reagent was removed, and then 150 μL of MTT solvent was added into each well to dissolve the deep purple formazan crystals. The tissue culture plate (TCP) was taken as a control. The absorbance values at 590 nm were measured by a microplate reader (Tecan, Grödig, Austria). To evaluate chondrocyte attachment on the surfaces of the fibrin beads, they were stained with phalloidin/DAPI, following the manufacturer’s protocol. The stained samples were visualized under a Zeiss laser scanning inverted confocal microscope and further processed using the software QuPath v3.0.0.

### 4.5. Cartilage Defect Localization and Bioactive Fibrin Implantation

**Figure 6:**
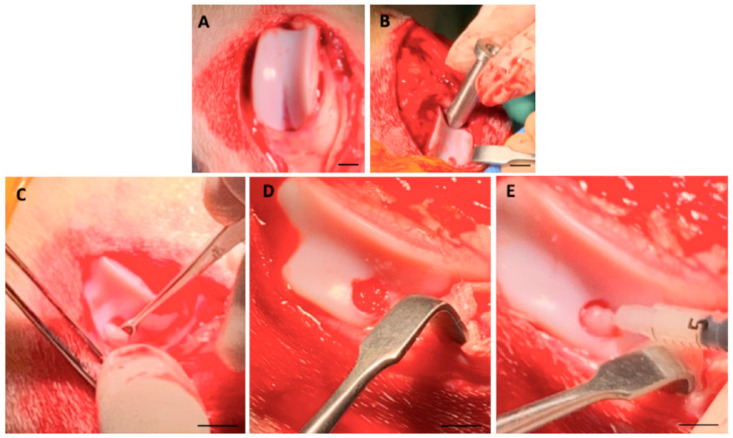
Surgical procedure for creation of a chondral defect and recombinant IGF-1-loaded fibrin microbead (FibIGF1) injection into the defect area (**A**) The left medial femoral condyle was exposed. (**B**) Two full-thickness chondral defects of 5 mm diameter and 2 mm depth were created using chondrotome (one not visible). (**C**) The cartilage was removed using a spatula. (**D**) The defect area was cleaned using saline. (**E**) FibIGF1 was injected into the defect area.

Following the administration of general anesthesia by intravenous ketamine (5 mg/kg) and diazepam (0.2 mg/kg) injection, the knee joints were surgically approached through medial parapatellar incisions under aseptic conditions [9]. The articular surface was exposed by laterally dislocating the patellae. By using a custom-made cylindrical chondrotome, a full-thickness chondral defect measuring 5 mm diameter and 2 mm depth was created on the weight-bearing region of the medial condyle of the left knee joint of each goat (Figure 6A,B). A similar defect was also created at a site in a non-weight-bearing region superior to the one previously created (patellofemoral region) (Figure 1C,D) and filled with Fib and FibIGF1, for Groups 1 and 2, respectively (Figure 1E).

### 4.6. Macroscopic Evaluation

At the 1st and 6th months after the various interventions were performed, all goats were sacrificed following the animal euthanasia procedures of the IACUC by severing the jugular vein located superficially at the neck of the animal. The knee joint was dissected to obtain the distal femur. The repaired chondral defects were visually inspected and histologically examined. Gross assessment of the repaired chondral defect area was performed by two independent observers blinded to the therapy, based on a modified component of the International Cartilage Repair Society (ICRS) Cartilage Repair Assessment scoring scale (macroscopic appearance subcategory) [61].

### 4.7. Histology

After gross inspection, the operative knees were harvested for histologic examination. The samples were fixed in 4% paraformaldehyde for 48 h. They were then rinsed in running tap water for 24 h and incubated with 10% formic acid for two months, followed by neutralization with 0.1% aqueous ammonia solution for 30 min. Decalcification was performed at room temperature under continuous shaking. After decalcification, the samples were dehydrated with a gradient ethanol series, washed extensively with xylene, and embedded in paraffin blocks. Cross-sections of 5 mm thickness were stained with hematoxylin and eosin (H&E), Safranin-O, and Alcian Blue, following standard procedures. Then, representative samples from each group were further immunostained with a polyclonal antibody against collagen II (II-II6B3, Developmental Studies Hybridoma Bank, Iowa City, IA, USA). Deparaffinized sections were pretreated with 1% hydrogen peroxide and incubated for 15 min with 2 mg/mL testicular hyaluronidase in PBS (pH 5) at 37 °C, followed by another 30 min with 1.5% normal goat serum and overnight at 4 °C with the primary antibody. After several washes with PBS, a secondary antibody of biotinylated horse anti-mouse IgG (Vector, Burlingame, CA, USA) was applied to the sections for 30 min at room temperature. Immunostaining was conducted with Vectastain ABC reagent (Vector, Burlingame, CA, USA) followed by 3,30-diaminobenzidine (DAB) staining. The mounted slides were imaged using an Olympus slide scanner. Cartilage histopathology scoring was also performed according to the modified O’Driscoll histological cartilage scoring system [62].

### 4.8. Statistical Analysis 

Unless otherwise mentioned, data are presented as mean ± standard deviation (SD). Means were compared using either two-tailed *t*-tests or one-way ANOVA tests, followed by Bonferroni correction for multiple comparisons. Statistical analyses were performed using GraphPad Prism (https://www.graphpad.com/features, accessed on 16 November 2023), with *p* < 0.05 as the criterion for statistical significance. 

## Data Availability

Data is contained within the article.

## Supplementary Materials

The following supporting information can be downloaded at: https://www.mdpi.com/article/10.3390/ijms242316945/s1.
